# Evaluation and comparison of hereditary Cancer guidelines in the population

**DOI:** 10.1186/s13053-021-00188-9

**Published:** 2021-07-17

**Authors:** Jordon B. Ritchie, Cecelia Bellcross, Caitlin G. Allen, Lewis Frey, Heath Morrison, Joshua D. Schiffman, Brandon M. Welch

**Affiliations:** 1 Medical University of South Carolina, 22 WestEdge St, Ste 200, Charleston, SC 29403 USA; 2 Emory University, Atlanta, USA; 3 ItRunsInMyFamily.com, Charleston, SC USA; 4 University of Utah, Salt Lake City, USA

**Keywords:** Clinical practice guidelines, Hereditary cancer, Family health history, Risk assessment

## Abstract

**Background:**

Family health history (FHx) is an effective tool for identifying patients at risk of hereditary cancer. Hereditary cancer clinical practice guidelines (CPG) contain criteria used to evaluate FHx and to make recommendations for genetic consultation. Comparing different CPGs used to evaluate a common set of FHx provides insight into how well the CPGs perform, the extent of agreement across guidelines, and how well they identify patients who should consider a cancer genetic consultation.

**Methods:**

We compare the American College of Medical Genetics and Genomics (ACMG) and the National Comprehensive Cancer Networks (NCCN) (2019) CPG criteria for FHx collected by a chatbot and evaluated by ontologies and web services in a previous study. Collected FHx met criteria from seven groups: Gene Mutation, Breast and Ovarian, Li-Fraumeni syndrome (LFS), Colorectal and Endometrial, Relative Meets Criteria, ACMG Only Criteria, and NCCN Testing. CPG Criteria were coded and matched across 12 ACMG sub-guidelines and 6 NCCN sub-guidelines for comparison purposes.

**Results:**

The dataset contains 4915 records, of which 2221 met either ACMG or NCCN criteria and 2694 did not. There was significant overlap—1179 probands met both ACMG and NCCN criteria. The greatest similarities were for Gene Mutation and Breast and Ovarian criteria and the greatest disparity existed among Colorectal and Endometrial criteria. Only 156 positive gene mutations were reported and of the 2694 probands who did not meet criteria, 90.6% of them reported at least one cancer in their personal or family cancer history.

**Conclusion:**

Hereditary cancer CPGs are useful for identifying patients at risk of developing cancer based on FHx. This comparison shows that with the aid of chatbots, ontologies, and web services, CPGs can be more efficiently applied to identify patients at risk of hereditary cancer. Additionally this comparison examines similarities and differences between ACMG and NCCN and shows the importance of using both guidelines when evaluating hereditary cancer risk.

## Background

### Family health history indicates risk for hereditary cancer

Family health history (FHx) is an effective tool for identifying individuals at risk for hereditary cancer [1–3]. An estimated 5–10% of all cancers are hereditary and individuals with a hereditary cancer syndrome are often at risk of developing cancer at a young age and more than one type [4–6]. Despite FHx being an effective way to estimate a patient’s risk of developing hereditary cancer, this important information is often not collected by clinicians resulting in many patients not being referred for genetic counseling. As many as 50% of patients with accurate FHx highly suggestive of hereditary risk for breast and ovarian cancer syndrome (HBOC) or Lynch syndrome (LS) are not being referred for genetic counseling [7, 8]. Additionally, an estimated 20% of primary care patients have family histories that indicate increased risk of developing a hereditary cancer [5, 9]. To improve overall cancer outcomes for patients and their families, it is important to conduct an effective evaluation of hereditary cancer clinical practice guidelines (CPG). The National Comprehensive Cancer Network (NCCN) and the American College of Medical Genetics and Genomics (ACMG) have each curated sets of CPGs based on FHx designed to support medical professionals in assessing FHx risk for hereditary cancer [10–12]. Each criterion in a CPG establishes thresholds based on elements of a patient’s FHx, such as number and types of cancers and their age of onset, to determine whether or not an individual is at risk of developing hereditary cancer and should consider receiving genetic counseling. Unfortunately, referral rates are low for cancer genetic consultations because providers lack training and confidence in assessing FHx for hereditary cancer risk [4, 13, 14]. Additionally, hereditary cancer CPGs are complicated and frequently updated. Another obstacle is that providers have insufficient time to collect and analyze FHx during a patient visit [15, 16].

### Collecting and evaluating patient FHx using chatbots, APIs, and ontologies

In 2019 we deployed an ad campaign which invited users to engage with a user-friendly chatbot designed to collect patient FHx. It reached over 14,000 participants, a quarter of which completed a full FHx [17]. The results demonstrated that chatbots are an effective tool for collecting FHx and that there is an interest in the population for understanding hereditary cancer risk. The chatbot eases the burden of collecting patient data associated with traditional surveys and webforms by allowing users to engage with a human-like texting interface. However, collecting FHx is only half the task and evaluating CPG criteria for FHx is challenging and time-consuming. Ontologies and web application programming interfaces (APIs) are used to assess patient FHx by formalizing the CPG criteria and providing access to them Ritchie JB, et al: Enabling Patients to Receive Clinical Practice Guideline Recommendations for Hereditary Cancer Risk Using Chatbots, Family History, Application Programming Interfaces (API), Ontologies, and Owlready2: System Description, under review. CPG criteria have two crucial information components: logical and clinical. Logical information, such as how many cancers run in a family and the age of onset for meeting a criteria, describe the thresholds that a patient’s FHx must meet in order to warrant a recommendation to consider genetic counseling. Clinical information, such as disease factors and distinguishing between different cancer subtypes, determines which thresholds are and are not met by a patient’s FHx. Ontologies are designed to represent this kind of knowledge in a machine-readable format so that the logical and clinical elements of CPG criteria can be understood together and interpreted by the computer. The chatbot can send a patient’s FHx to the ontology through web APIs, which apply CPG criteria and return the appropriate recommendations. Recommendations returned to the patient can also be sent to their provider to determine whether a cancer genetic consultation should be considered. Chatbots combined with web-based, ontology-oriented programming not only provide patients access to understanding CPG criteria and the implications they might have for their health with respect to cancer, but also can alleviate the strain on clinicians to collect and analyze FHx during patient visits.

This study reports on referral recommendations for 4915 users who completed the ItRunsInMyFamily chatbot workflow and received a recommendation to consider a genetics consultation (positive screen) or not (negative screen) based on CPG criteria [17]. The primary aim of this report is to understand how many of these 4915 patients met criteria, which criteria they met, how many received recommendations to receive a cancer genetic consultation, what types of cancers they had, and which genetic mutations were reported. For probands who screen negative, we also report those with a history of cancer in their family. The secondary aim of this study is to provide a side-by-side comparison of ACMG and NCCN hereditary cancer CPGs with respect to the collected patient FHx. Characterizing the patients who engaged the chatbot and collected their FHx demonstrates the utility of chatbots, ontologies, and web services in assessing hereditary cancer risk. Comparing ACMG and NCCN highlights agreement and disparity between the two CPGs as well as provides insight into the percentages and types of cancer criteria that are met by the patients.

## Methods

### Data collection and analysis

In November 2019, Welch et al. ran an online marketing campaign that ultimately collected FHx for 4915 users [17]. The majority of users during this campaign were 40–59 year old women and about half reported European ancestry. On average, patients had just under 20 family members in their family history. Welch et al. provide an in-depth analysis of the patient cohort including lifestyle (smoking, alcohol consumption, etc.), screening (mammogram, colonoscopy, etc.), and other personal and family health history metrics. In the present study, we considered only patients whose full FHx had been collected and performed descriptive analysis in Google Sheets to compare the frequency and type of criteria met for ACMG and NCCN CPGs.

We used criteria from the ACMG 2015 guideline; the Genetic/Familial High-Risk Assessment: Breast and Ovarian, Version 1.2020; and the Genetic/Familial High-Risk Assessment: Colorectal Version 3.2019, NCCN Clinical Practice Guidelines in Oncology [10–12]. Not all criteria represented in the ontology are included in this analysis—just those met by patients based on their FHx.

To compare and evaluate criteria from both ACMG and NCCN guidelines, we coded and numbered the criteria met by probands for each guideline included in the hereditary cancer ontology used to assess FHx. For the purposes of this paper, we divided each guideline into sub-guidelines to enable comparison of similar sets of criteria. ACMG criteria from 12 sub-guidelines—SyndromePatient, Brain, Breast, Ovarian, Colorectal, Endometrial, Leukemia, Gastric, Prostate, Melanoma, Thyroid, and AtRiskFDR—and NCCN criteria from six sub-guidelines—CRIT1, CRIT4, HRS3, POLYP1, LS1, and GENE1—were met by patients. Individual criteria are coded based on the sub-guidelines they are from and the order of their sequence in said sub-guideline e.g. Breast.01, Breast.02 for the first two criteria in the ACMG Breast sub-guideline. The SyndromePatient sub-guideline criteria refer to patients with a positive personal or familial gene mutation and AtRiskFDR sub-guideline criteria refer to patients at risk because a family member meets ACMG criteria. CRIT1, CRIT4, and GENE1 are sub-guidelines of the NCCN Genetic/Familial High-Risk Assessment: Breast, Ovarian, and Pancreatic guideline and include criteria covering breast cancer, ovarian cancer, pancreatic cancer, positive gene mutations, LFS, and risk conveyed by FDRs who meet criteria. HRS3, POLYP1, and LS1 are sub-guidelines of the NCCN Genetic/Familial High-Risk Assessment: Colorectal guideline and include criteria covering colorectal cancer, endometrial cancer, polyps, and LS.

**Fig. 1:**
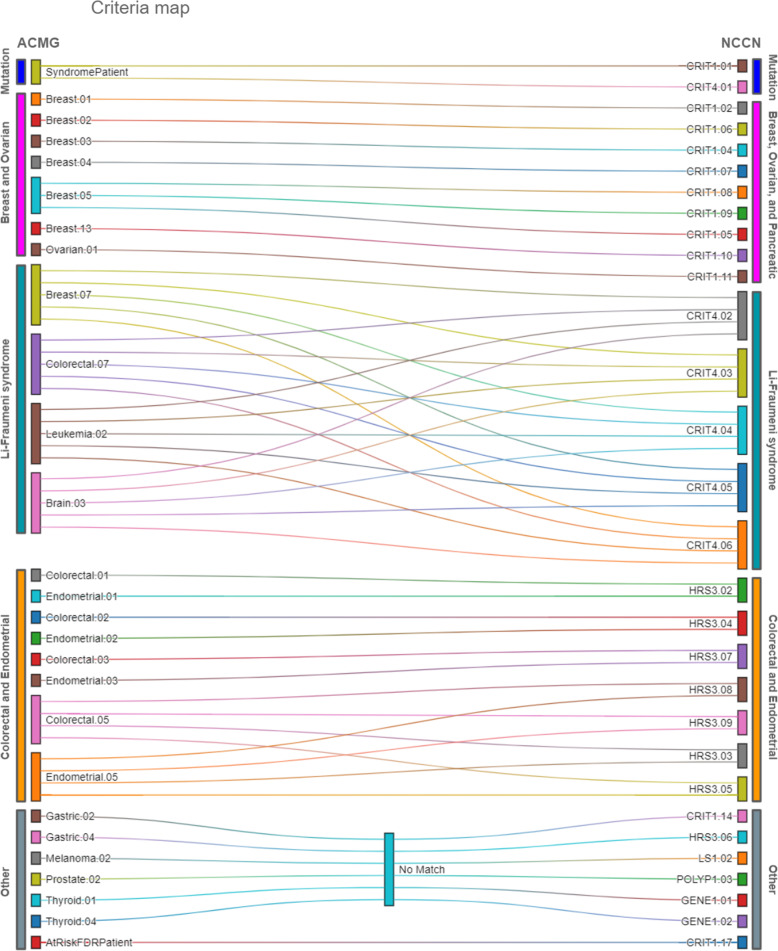
Criteria map. ACMG coded criteria mapped to NCCN coded criteria based on similar or matching clinical information and thresholds. Criteria codes are named for the sub-guideline they come from and the order with which they appear in the sub-guideline e.g. ACMG Breast.01 is the first criteria in the breast cancer sub-guideline in ACMG and CRIT1.01 is the first criterion in the breast, ovarian, and pancreatic guidelines sub-guideline CRIT1 in NCCN. ACMG (American College of Medical Genetics and Genomics); NCCN (National Comprehensive Cancer Network)

**Fig. 2:**
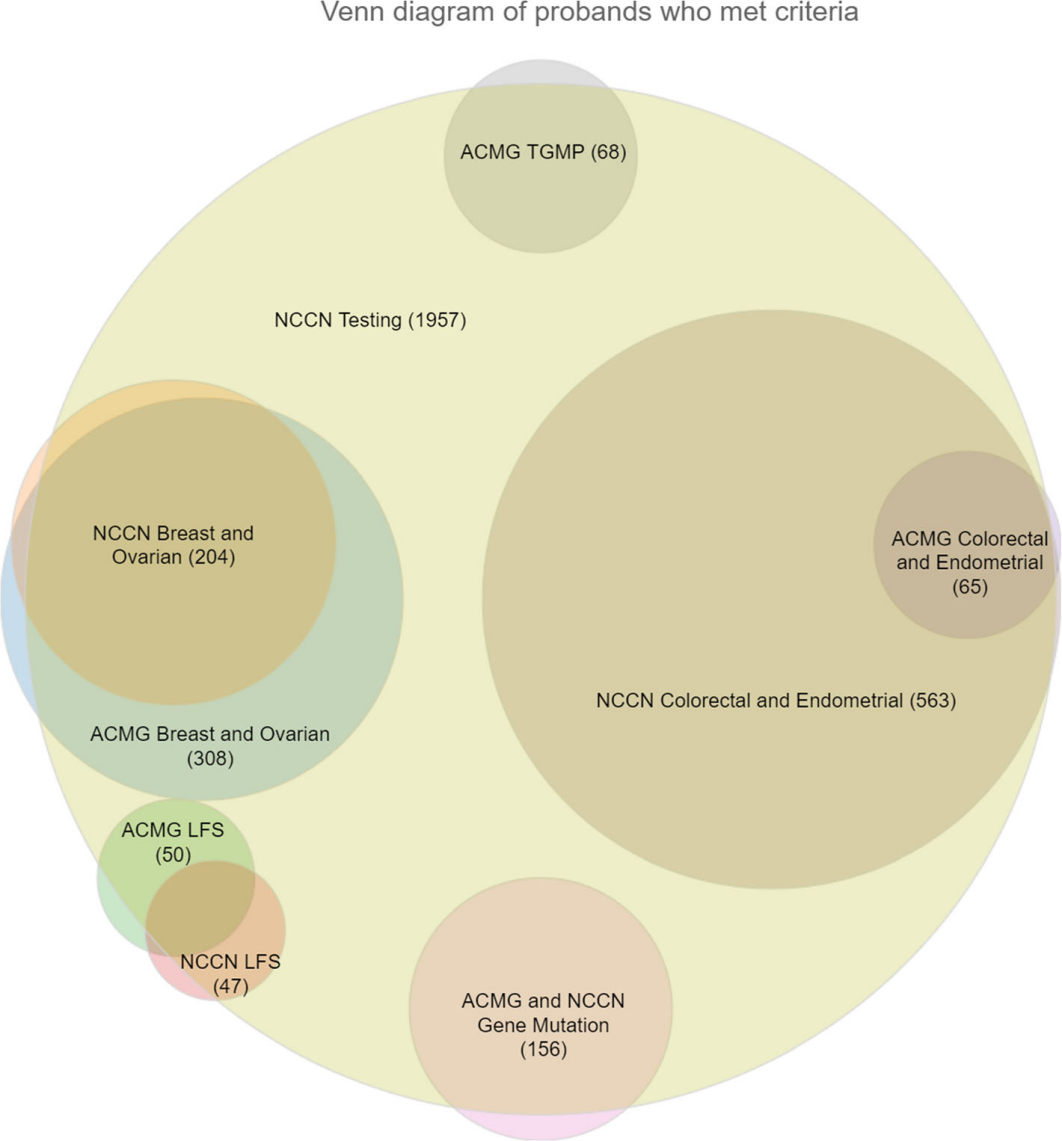
Venn diagram of probands who met criteria.This Venn diagram displays analogous ACMG and NCCN criteria groups met by probands. The relative size of each circle represents the number of probands who met criteria in the respective group. TGMP (thyroid, gastric, melanoma, prostate); LFS (Li-Fraumeni syndrome); ACMG (American College of Medical Genetics and Genomics); NCCN (National Comprehensive Cancer Network)

Figure 1 maps coded criteria from ACMG and NCCN guidelines that are either word for word the same or include the same clinical elements with slightly different thresholds. ACMG Breast.07, Colorectal.07, Leukemia.02, and Brain.03 represent ACMG LFS criteria and are each mapped to the corresponding group of NCCN LFS criteria CRIT4.02–06. Criteria in ACMG and NCCN with no sufficiently similar counterpart in the other CPG are mapped to No Match. We grouped matching or similar criteria into the following groups: Gene Mutation; Breast and Ovarian; LFS, Colorectal and Endometrial; Relative Meets Criteria; ACMG Thyroid, Gastric, Melanoma, Prostate (TGMP); and NCCN Testing (Fig. 2). In many cases, a criterion was common across both guidelines. By matching similar criteria across ACMG and NCCN into specific groups, we were able to compare patients who met criteria from one, both, or neither guideline.

## Results

Of the patients who engaged with the chatbot to collect their FHx, 4915 completed the chatbot workflow and received a risk assessment report. Of these, 2221 (45.2%) met ACMG or NCCN criteria for a genetic cancer consultation referral and 2694 (54.8%) did not meet criteria. Twenty-four percent (1179/4915) met both ACMG and NCCN criteria for a genetic cancer consultation. Only 765 (15.6%) met NCCN criteria and not ACMG criteria while 277 (5.6%) patients met AMG criteria and not NCCN criteria.

Probands who met criteria often met criteria from more than one sub-guideline. Figure 2 shows the main overlaps among several of the different groups of guideline criteria we studied. Not all overlapping criteria groups could be displayed because the full set of overlapping CPGs was impossible to render as a Venn diagram. Instead, homologous criteria groups for both CPGs were intersected, and all groups were intersected with NCCN testing. The majority of probands who met criteria that recommended genetic counseling from either ACMG or NCCN met an NCCN criteria that recommended genetic testing as well (1957/2221; 88%). Tables 1 and 2 contain the codes, criteria text, and the number of probands that met individual criteria and overall sub-guidelines.

**Table 1:** NCCN coded criteria

Code	Criteria	Totals
**Gene Mutation (CRIT1, CRIT4)**		**156**
CRIT1.01	Individuals with any blood relative with a known pathogenic/likely pathogenic variant in a cancer susceptibility gene	156
CRIT4.01	Individual from a family with a known TP53 pathogenic/likely pathogenic variant	15
**Breast, Ovarian and Pancreatic (CRIT1)**		**202**
CRIT1.02	Breast cancer dx at age ≤ 45	70
CRIT1.06	Breast cancer dx at age ≤ 60 y with triple-negative breast cancer	17
CRIT1.04	Breast cancer dx at age 46-50y with a second breast cancer dx at any age	1
CRIT1.07	Breast cancer dx at any age with Ashkenazi Jewish ancestry	7
CRIT1.08	Breast cancer dx at any age with ≥1 close blood relative with breast cancer at age ≤ 50 y or ovarian pancreatic or metastatic or intraductal prostate cancer at any age	49
CRIT1.09	≥3 total diagnoses of breast cancer in patient and/or close blood relatives	72
CRIT1.05	Breast cancer dx at age 46-50y with ≥1 close blood relative with breast, ovarian, pancreatic, or high-grade (Gleason score ≥ 7) or intraductal prostate cancer at any age	17
CRIT1.10	Diagnosed at any age with male breast cancer	1
CRIT1.11	Epithelial ovarian cancer (including fallopian tube cancer or peritoneal cancer) at any age	38
CRIT1.14	High-grade (Gleason score ≥ 7) prostate cancer with Ashkenazi Jewish ancestry	2
**Li-Fraumeni Syndrome (CRIT4)**		**47**
CRIT4.02	Individual diagnosed at age < 45 y with Non Ewing Sarcoma AND a first degree relative diagnosed at age < 45 y with cancer AND an additional first- or second-degree relative in the same lineage with cancer diagnosed at age < 45 yor a sarcoma at any age	2
CRIT4.03	Individual with a tumor from LFS tumor spectrum (eg soft tissue sarcoma, osteosarcoma, CNS tumor, breast cancer, adrenocortical carcinoma) before 46 y of age AND at least one first- or second-degree relative with any of the aforementioned cancers (other than breast cancer if the proband has breast cancer) before the age of 56 y or with multiple primaries at any age	34
CRIT4.04	Individual with multiple tumors (except multiple breast tumors)two of which belong to LFS tumor spectrum with the initial cancer occurring before the age of 46 y	9
CRIT4.05	Individual with adrenocortical carcinoma or choroid plexus carcinoma or rhabdomyosarcoma of embryonal anaplastic subtype at any age of onset regardless of family history	1
CRIT4.06	Breast cancer before 31 y of age	10
**Colorectal and Endometrial (HRS3)**		**563**
HRS3.02	Colorectal or endometrial cancer diagnosed < 50 y	59
HRS3.04	An individual with colorectal or endometrial cancer and ≥ 1 first-degree or second-degree relative with LS-related cancer diagnosed < 50 y	17
HRS3.07	≥1 first-degree relative with colorectal or endometrial cancer and another synchronous or metachronous LS-related cancer	90
HRS3.08	≥2 first-degree or second-degree relatives with LS-related cancers including ≥1 diagnosed < 50 y	296
HRS3.09	≥3 first-degree or second-degree relatives with LS-related cancers regardless of age	160
HRS3.03	An individual with colorectal or endometrial cancer and another synchronous or metachronous LS-related cancer	6
HRS3.05	An individual with colorectal or endometrial cancer and ≥ 2 first-degree or second-degree relative with LS-related cancers regardless of age	10
HRS3.06	≥1 first-degree relative with colorectal or endometrial cancer diagnosed < 50 y	174
**Relative meets criteria (CRIT1)**		**1662**
CRIT1.17	An affected or unaffected individual with a first- or second-degree blood relative meeting any of the NCCN Genetic/Familial High-Risk Assessment: Breast Ovarian and Pancreatic personal risk criteria from CRIT-1	1662
**NCCN testing (LS1, POLYP1, GENE1)**		**1957**
LS1.02	Meets HRS-3 criteria but no known pathogenic/likely pathogenic variant in proband or family	562
POLYP1.03	No known pathogenic variants in any polyposis gene in family and meets POLYP1.01 criteria	47
GENE1.01	Meets NCCN Genetic/Familial High-Risk Assessment: Breast Ovarian and Pancreatic criteria from CRIT-1, CRIT-2, CRIT-4, or CRIT-5 and familial pathogenic/likely pathogenic variant known	96
GENE1.02	Meets NCCN Genetic/Familial High-Risk Assessment: Breast Ovarian and Pancreatic criteria from CRIT-1, CRIT-2, CRIT-4, or CRIT-5 but no known familial pathogenic/likely pathogenic variant	1606

These are the criteria included in the hereditary cancer ontology used to evaluate FHx from NCCN that at least one proband met. Each section (Gene Mutation, LFS, Colorectal and Endometrial, Relative meets criteria, and NCCN testing) groups related criteria together. For each criterion in a section the total number of probands who met that criteria are recorded. Given that a proband can meet more than one criteria, the section totals are the total number of probands who met at least one criteria from the criteria in that section

**Table 2:** ACMG coded criteria

Code	Criteria	Totals
**Gene Mutation**		**156**
ACMGSyndromePatient	Has known mutation in cancer susceptibility gene	156
**Breast and Ovarian**		**308**
Breast.01	Breast cancer dx at age ≤ 50	90
Breast.02	Triple-negative breast cancer dx at age ≤ 60	17
Breast.03	≥2 primary breast cancers in the same person	3
Breast.04	Ashkenazi Jewish ancestry and breast cancer at any age	7
Breast.05	≥3 cases of breast, ovarian, pancreatic, and/or aggressive prostate cancer in close relatives including the patient	202
Breast.13	Single case (male breast cancer) present	1
Ovarian.01	Single case (ovarian, fallopian tube, or primary peritoneal cancer) present in the patient or a FDR	38
**Li-Fraumeni Syndrome**		**50**
Breast.07	Breast cancer and one additional LFS tumor in the same person or in two relatives one dx at age ≤ 45	39
Colorectal.07	Colorectal cancer and one additional LFS tumor in the same person or in two relatives one dx at age ≤ 45	11
Leukemia.02	Leukemia and one additional LFS tumor in the same person or in two relatives one dx at age ≤ 45	1
Brain.03	Brain tumor and one additional LFS tumor in the same person or in two relatives one dx at age ≤ 45	2
**Colorectal and Endometrial**		**65**
Colorectal.01	Colorectal cancer dx at age < 50	21
Endometrial.01	Endometrial cancer dx at age < 50	39
Colorectal.02	Colorectal cancer dx at age ≥ 50 if there is a FDR with colorectal or endometrial cancer at any age	4
Endometrial.02	Endometrial cancer dx at age ≥ 50 if there is a FDR with colorectal or endometrial cancer at any age	1
Colorectal.03	Synchronous or metachronous colorectal or endometrial cancers in the same person	1
Endometrial.03	Synchronous or metachronous colorectal or endometrial cancer in the same person	1
Colorectal.05	Colorectal cancer and two additional cases of any LS-associated cancer in the same person or in close relatives	2
Endometrial.05	Endometrial cancer and 2 additional cases of any LS-associated cancer in the same person or in close relatives	9
**Relative meets criteria**		**1306**
ACMGAtRiskFDRPatient	First degree relative (mother father or sibling) meets risk criteria	1306
**Remaining ACMG**		**68**
Gastric.02	≥2 cases of gastric cancer one dx at age < 50 in close relatives	9
Gastric.04	≥3 cases of gastric cancer in close relatives	1
Melanoma.02	≥3 cases of melanoma and/or pancreatic cancer in close relatives	3
Prostate.02	≥2 cases of prostate cancer dx at age ≤ 55 in close relatives	1
Thyroid.01	Medullary thyroid cancer	11
Thyroid.04	Papillary thyroid cancer (cribriform-morular variant)	46

These are the criteria included in the hereditary cancer ontology used to evaluate FHx from ACMG that at least one proband met. Each section (Gene Mutation, LFS, Colorectal and Endometrial, Relative meets criteria, and Remaining ACMG) groups related criteria together. For each criterion in a section the total number of probands who met that criteria are recorded. Given that a proband can meet more than one criteria, the section totals are the total number of probands who met at least one criteria from the criteria in that section

Of the 4915 patients who entered their FHx, 1969 (40.1%) met NCCN criteria of which 1957 (99.4%) met NCCN Testing criteria, 204 (10.4%) patients met Breast, Ovarian, and Pancreatic CRIT1, 47 (2.4%) met Breast, Ovarian, and Pancreatic CRIT4, and 563 (28.6%) met Colorectal HRS3. The 12 patients who met NCCN criteria but did not receive a recommendation for genetic testing had already reported a positive genetic mutation in their family and therefore did not receive a recommendation for testing per NCCN guidelines. However, they did receive a recommendation to consider a cancer genetic consultation per ACMG and NCCN. Of the 1969 patients who met NCCN criteria, a total of 1662 (84.4%) had FHx that met NCCN criteria for counseling or testing, and 833 (42.3%) had personal cancer history that met NCCN criteria for counseling or testing. Twenty-eight percent of the 1969 patients who met NCCN criteria had personal and FHx that met NCCN criteria for genetic counseling or testing.

Nearly a third of the 4915 patients who collected their FHx—1456 (29.6%)—met ACMG criteria for a cancer genetics consultation. ACMG has criteria only for cancer genetic consultations and none for genetic testing. Of the 1456 patients who met ACMG criteria, 308 (21.2%) met criteria for ACMG breast and ovarian, 65 (4.5%) met ACMG criteria for colorectal and endometrial, 50 (3.4%) met ACMG LFS-related criteria, and 68 (4.7%) met ACMG criteria for gastric, melanoma, prostate, or thyroid cancer. Nearly 90% (1306/1456; 89.7%) had FHx sufficient to warrant a recommendation for a cancer genetic consultation and 545 (37.4%) had sufficient personal cancer history to warrant a recommendation for a cancer genetic consultation. Just over 15 % (225/1456; 15.5%) had both personal and FHx sufficient to meet criteria for a cancer genetic consultation recommendation.

Additionally, of the 4915 patients who collected their FHx, 156 (3.2%) patients met both the NCCN and ACMG criteria for receiving a genetic counseling recommendation based on reported positive genetic test results in their FHx. In total, 621 (12.6%) genetic test results were reported. Personal genetic tests accounted for 258 (41.5%) of those of which 60 (23.3%) were positive test results, 160 (62.0%) were negative test results, and 38 (14.7%) were test results where the patient reported having a test but did not indicate the result. Genetic tests for 363 (363/621; 58.5%) family members accounted for the rest with 132 (36.4%) positive test results, 129 (35.5%) negative test results, and 102 (28.1%) unknown test results. Twenty (3.2%) probands reported a personal genetic test result and a genetic test result for a family member; and 8 (1.3%) probands reported a personal genetic test result and genetic test results for two or more family members.

**Fig. 3:**
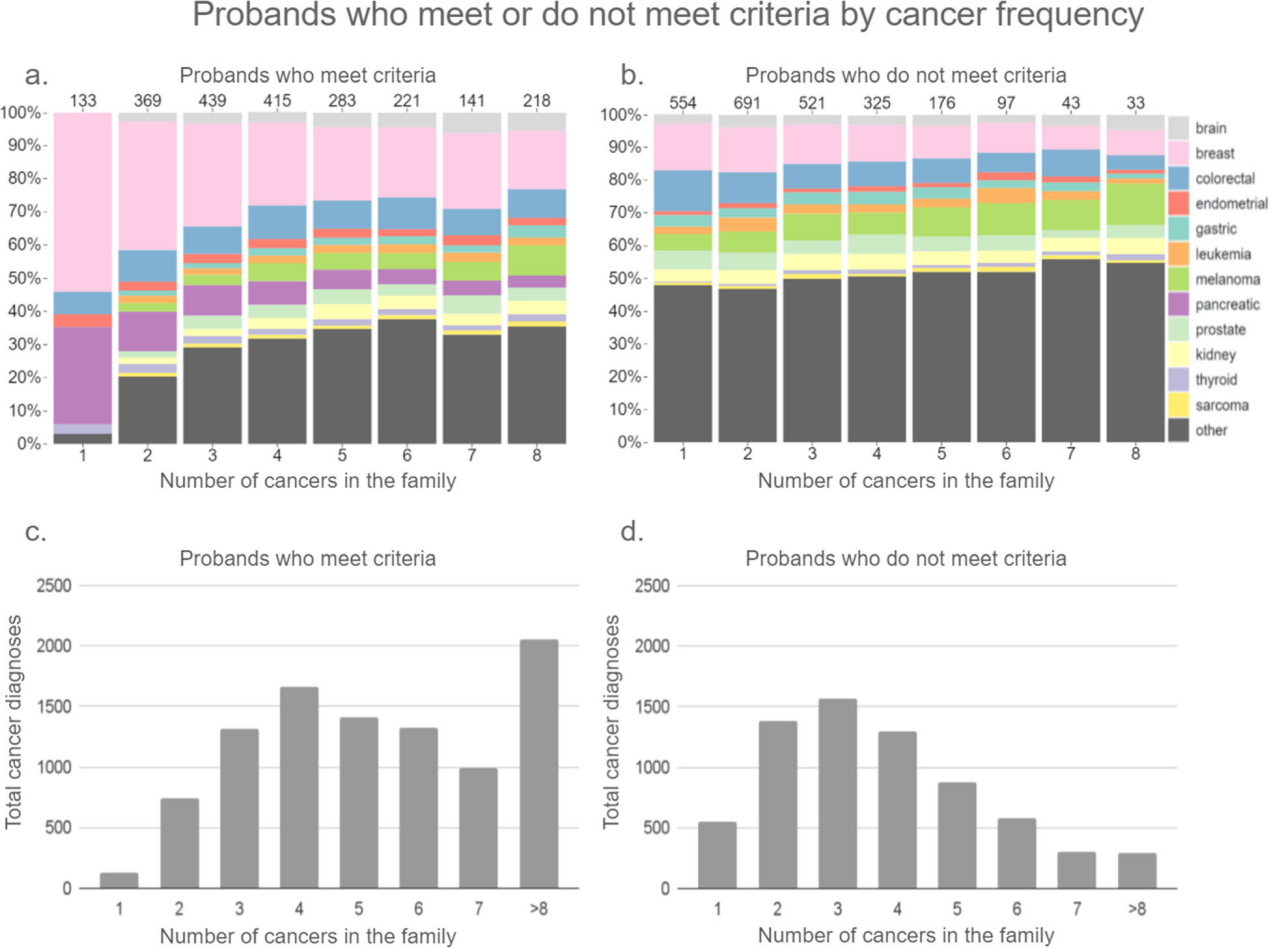
Probands who met or do not meet criteria by cancer frequency. **a**–**b** Shows the proportions of cancer types by the number of cancer diagnoses within individual family histories that did and did not meet criteria. **c**–**d** Shows the total number of cancer diagnoses for each corresponding cancer count for all family histories in that category

Of the 2694 patients who did not meet CPG criteria, 2440 (90.6%) had at least one cancer in their FHx. Of those 554 (22.7%) patients had one cancer in their family, 691 (28.3%) had two cancers, 521 (21.4%) had three, 325 (13.3%) had four, 176 (7.2%) had five, 97 (4.0%) had six, 43 (1.8%) had seven, and 33 (1.4%) had eight or more cancers in their FHx. Other cancers accounted for approximately half of the cancers reported by probands who had cancer in their family but did not meet criteria. However, as the number of cancers in the family increased for probands who did not meet criteria, the total number of probands decreased (Fig. 3d) indicating that families with higher instances of cancer are more likely to meet criteria despite reporting ‘other’ cancer types. Of the 2221 patients who did meet CPG criteria, 133 (6.0%) had one cancer in their family, 369 (16.6%) had two cancers, 439 (19.8%) had three, 415 (18.7%) had four, 283 (12.7%) had 5, 221 (10.0%) had 6, 141 (6.3%) had 7, and 218 (9.8%) had eight or more cancers in their FHx (Fig. 3). Breast cancer accounted for the largest proportion of cancers for which CPG criteria was implemented regardless of the number of cancers in the family. There were four probands with one ‘other’ cancer in their family who met criteria because a family member reported a positive cancer gene mutation. More families with three or fewer cancers did not meet CPG criteria than those that did, and more families with four or more cancers met criteria than those that did not (Fig. 3).

## Discussion

Using a chatbot, web APIs, and hereditary cancer ontologies, we collected complete FHx for 4915 probands and coded and analyzed the hereditary cancer CPG criteria they met from ACMG and NCCN. Collecting FHx for at-risk patients and applying hereditary cancer guidelines allowed us to examine performance of ACMG and NCCN in identifying patients at risk and highlight important differences and similarities among them (Fig. 2). Genetic Mutation, Breast and Ovarian, and LFS guideline criteria groups for ACMG and NCCN captured similar sized sets of probands. Genetic Mutation criteria was exactly the same for ACMG and NCCN and therefore captured identical sets of probands. ACMG captured a moderately larger set of probands than NCCN for Breast and Ovarian criteria and the set of probands who met NCCN Breast and Ovarian criteria were almost completely subsumed by the set of probands who met ACMG Breast and Ovarian criteria. LFS criteria captured similar-sized sets of probands for both guidelines but had less overlap than did Breast and Ovarian criteria. Probands who met Colorectal and Endometrial criteria had the greatest disparity. NCCN Colorectal and Endometrial guideline criteria captured a much larger set of probands and almost completely subsumed the set of probands who met the analogous ACMG criteria. NCCN had no analog to ACMG Thyroid, Gastric, Melanoma, or Prostate cancer criteria and NCCN alone had genetic testing criteria which vastly subsumed the majority of probands who met ACMG or NCCN criteria.

### Guideline comparison

ACMG and NCCN share overlap in guideline criteria with minor differences. The biggest difference between the two is ongoing updates for NCCN whereas ACMG was published in 2015 and has not been revised since. Additionally, ACMG includes criteria for a number of cancers that NCCN does not. These differences are strengths and weaknesses that influence how effectively the guidelines identify patients at risk. Frequent updates to NCCN keep the guideline criteria current, and ACMG catches patients at risk for cancers that NCCN does not publish criteria for. Together they balance each other in providing the best indication of risk for a patient based on their FHx.

### Breast and ovarian

Not surprisingly, the criteria groups with the most congruent criteria were Breast and Ovarian cancer (Fig. 1). ACMG and NCCN each had a single criterion for Ovarian, which, though worded differently, effectively states that any history of personal or familial ovarian, fallopian tube, or peritoneal cancer meets the criterion. Breast cancer criteria had very close mapping between ACMG and NCCN. Primary differences were in the thresholds and cancers that made up the logical component of the criteria. The differences that resulted in notable disagreement between guidelines include an earlier age of onset of breast cancer ≤45 in NCCN vs ≤50 in ACMG (CRIT1.01 vs Breast.01) accounting for 20 extra probands meeting ACMG criteria. The biggest difference is accounted for by ACMG Breast.05 (compare with NCCN CRIT1.05, 07, and 08) which says ≥3 cases of breast, ovarian, pancreatic, and/or aggressive prostate cancer in close relatives including the patient. The analogous NCCN Breast and Ovarian guideline criteria include age of onset restrictions, which significantly restrict the window for meeting the criteria (CRIT1.08–09) (Table 1). Additionally, NCCN restricts the set of cancers required to meet the criteria, resulting in fewer probands meeting the criteria (CRIT1.09) and accounting for why ACMG captured approximately 100 more probands than NCCN. Despite this difference, the Breast and Ovarian guideline criteria still bore the closest resemblance in content and thresholds other than the Gene Mutation guideline criteria.

### LFS

LFS guideline criteria identified similar-sized sets of patients but had less overlap than Breast and Ovarian criteria. The lack of overlap makes sense because the LFS criteria between ACMG and NCCN guidelines had the least in common in terms of wording and structure in their respective CPGs. LFS criteria from ACMG come from four separate sub-guidelines—Breast, Colorectal, Leukemia, and Brain—and all have the same text with the respective cancer substituted in. The NCCN CRIT4 sub-guideline is dedicated to LFS and has highly specific sets of criteria that cover all LFS cancers. Despite these differences, the sets of criteria displayed considerable overlap in their respective sets of probands.

### Colorectal and endometrial

Sets of probands identified by the Colorectal and Endometrial guideline criteria represented the greatest disparity in size between ACMG and NCCN. Like Breast and Ovarian, the Colorectal and Endometrial criteria for NCCN and ACMG were similar in wording and structure with a few exceptions that account for the large difference in the number of probands captured by these criteria (Fig. 1) (Tables 1 and 2). HRS3.06, which states: ≥1 first-degree relative with colorectal or endometrial cancer diagnosed < 50 y, has no analog in ACMG and accounts for 174 probands that meet NCCN Colorectal and Endometrial criteria. ACMG Colorectal and Endometrial criteria 02–03 have age of onset and range of family member restrictions that significantly affect the number of probands that meet the criteria. Comparatively, analogous NCCN criteria, HRS3.04 and HRS3.07, have looser restrictions on age of onset and an expanded range of family members who can meet the criteria respectively. The looser thresholds for these NCCN criteria account for almost 100 probands who meet criteria compared to less than 10 probands who meet analogous ACMG criteria. Probands met twice as many LS associated criteria in NCCN (HRS3.03, 05, 08, and 09) than they did in ACMG (Colorectal.05 and Endometrial.05). Additionally, NCCN LS criteria had lighter restrictions on cancers and a wider range of relatives that contributed to the logical thresholds required to meet criteria, resulting in over 400 probands who met NCCN criteria compared to about 10 probands who met analogous ACMG criteria (Tables 1 and 2). These two sets of Colorectal and Endometrial criteria from ACMG and NCCN demonstrate how very small changes in thresholds can have enormous effects on which patients meet criteria and receive recommendations to consider genetic counseling and testing.

### Thyroid, gastric, melanoma, and prostate

ACMG Thyroid, Gastric, Melanoma, and Prostate (TGMP) sub-guidelines have no obvious analog in NCCN for the criteria we implemented. However, 69 probands met criteria from one of these four sub-guidelines. The majority of them met Thyroid cancer criteria for medullary or papillary thyroid cancer. These cancers, and others, are less commonly focused on in the hereditary cancer guidelines but should not be forgotten.

### NCCN testing

The vast majority of patients who met either ACMG or NCCN criteria for genetic counseling also received a genetic testing recommendation from NCCN. It should be noted that ACMG did not have analogous genetic testing recommendations, only counseling. Importantly, less than one-tenth (156/2221; 7.0%) of patients who met criteria for hereditary cancer risk had received a genetic test. This statistic underscores the urgency for more effectively identifying those at risk for cancer based on FHx.

### Screened negative but had family history of cancer

The relatively high prevalence of cancers designated as “other” in this set of probands makes sense because these cancers would have little impact when evaluating guidelines for specific hereditary cancers. Indeed the guideline criteria from either ACMG or NCCN can say little about a family’s hereditary cancer risk based on, for example, two unknown cancer diagnoses in family members. The logical components of the CPGs are ineffective without the clinical components provided by the patient. Probands with breast cancer, colorectal cancer, and/or melanoma account for the next largest trends of probands with cancer prevalent in their FHx that did not meet CPG criteria. For families with large numbers of cancer for which specific guideline criteria are implemented, either age of onset was too great or cancers were shared across both sides of the family in such a way as to fail to meet criteria.

### Limitations and future directions

This study shows that FHx can be collected with user-friendly chatbots and that web services can provide access to risk assessment based on CPG criteria. We had a high screen positive rate of 45.2% which was likely due to participation bias. The dataset utilized here was collected via an ad campaign [17] and focused primarily on middle-aged women. Therefore, the results are skewed towards cancers more commonly found in women from that age group.

Eligibility for genetics consultation and meeting clinical practice criteria are not perfectly aligned. The primary purpose of this study was to determine the criteria met and the patterns of cancer in patients who recorded their FHx. There very likely are patients who do not strictly meet criteria but are probably eligible for genetic consultation and testing. However, evaluating the suitability of the clinical practice guidelines for accurately identifying the patients who are eligible for counseling and testing lies outside the scope of this study. Furthermore, not every sub-guideline and criteria from ACMG and NCCN was implemented and not every implemented sub-guideline and criteria is represented in this analysis. Because the primary aim of this study was to demonstrate the feasibility of automating application of CPG criteria to probands with potential risk of hereditary cancer conveyed by reported FHx, only the sub-guidelines and criteria that probands met were included in the analysis. Therefore, the criteria met by probands reflects the demographic surveyed by skewing towards cancers most common in middle-aged women.

## Conclusion

The primary aim of this research study was to demonstrate the feasibility of automating hereditary cancer risk assessment using chatbots, ontologies, and web services by comparing patients who participated and the CPG criteria they met. These results show hereditary cancer CPGs can be automated using chatbots and ontology-driven risk assessment. Additionally, the secondary aim of this research study was to provide a side-by-side comparison for ACMG and NCCN hereditary cancer CPGs. ACMG and NCCN Breast and Ovarian guideline criteria are the most similar across all criteria we compared, with the exception of the Gene Mutation criteria, which is an exact match. Colorectal and Endometrial guideline criteria have the greatest disparity in the number of probands who met these criteria, showing that despite their similar wording and structure, small changes to thresholds—including cancers and the range of family members the criteria apply to—can vastly affect the number of patients who meet criteria and receive recommendation to consider genetic counseling/testing consultation. Overall, probands who met criteria generally met more than one and often met criteria from multiple sub-guidelines within ACMG and NCCN.

Identifying patients who meet criteria from these guidelines is challenging and nuanced, but with the help of technological solutions, this burden can be lifted from off the shoulders of frontline care providers. More efficient and effective identification of patients who should consider genetic counseling and testing consultations will lead to earlier interventions and preventive measures that can improve outcomes among individuals at risk for hereditary cancer.

## Data Availability

The datasets used and/or analysed during the current study are available from the corresponding author on reasonable request.

## Abbreviations

FHx: Family health history
HBOC: Hereditary breast and ovarian cancer syndrome
LS: Lynch Syndrome
CPG: Clinical practice guidelines
NCCN: National Comprehensive Cancer Network
ACMG: American College of Medical Genetics and Genomics
API: Application programming interface
TGMP: Thyroid, gastric, melanoma, and prostate
